# Mortality associated with ingestion of sea urchins in loggerhead sea turtles (*Caretta caretta*): A case series

**DOI:** 10.1371/journal.pone.0221730

**Published:** 2019-08-23

**Authors:** Alicia Inurria, Alberto Arencibia, Pascual Calabuig, May Gómez, Soraya Déniz, Jorge Orós

**Affiliations:** 1 Department of Morphology, Veterinary Faculty, University of Las Palmas de Gran Canaria (ULPGC), Arucas (Las Palmas), Spain; 2 Tafira Wildlife Rehabilitation Center, Tafira Baja-Las Palmas de Gran Canaria, Spain; 3 Marine Ecophysiology Group (EOMAR), University Institute for Sustainable Aquaculture and Marine Ecosystems (IU-ECOAQUA), University of Las Palmas de Gran Canaria (ULPGC), Telde (Las Palmas), Spain; 4 Unit of Infectious Diseases, Veterinary Faculty, University of Las Palmas de Gran Canaria (ULPGC), Arucas (Las Palmas), Spain; Faculty of Animal Sciences and Food Engineering, University of São Paulo, BRAZIL

## Abstract

**Aims:**

The aims of this study were: a) to describe the pathological and laboratory findings in a case series of stranding and mortality associated with ingestion of large amounts of sea urchins in loggerhead turtles (*Caretta caretta*), and b) to alert veterinarians and biologists involved in sea turtle conservation of this cause of stranding and/or death.

**Methods:**

The six loggerheads studied were stranded on the coasts of Gran Canaria, Canary Islands, Spain, between 2008 and 2015. Post mortem studies included pathological, microbiological, and sea urchin species identification procedures.

**Results:**

All turtles showed severe intestinal impaction caused by large amounts of sea urchins, mainly affecting the colon and the caudal half of the small intestine. Histologically, severe focal fibrinonecrotic enteritis was diagnosed in two turtles. In the remaining turtles, lesions ranged from mild desquamation of the intestinal epithelium to severe congestion of the blood vessels of lamina propria, submucosa, muscular and serosa, and edema. *Vibrio* sp. was isolated from the spleen and intestinal mucosa of a loggerhead in which focal fibrinonecrotic enteritis had been diagnosed. In five turtles, all the remains were fragments from long-spined sea urchins (*Diadema africanum*); the last turtle contained a mixture of long-spined sea urchin (90%) and purple sea urchin (*Sphaerechinus granularis*) (10%) remains.

**Conclusions:**

Although the prevalence of this cause of stranding was low (< 1.6%) compared to other mortality causes, continued overfishing and anthropogenic climate change could increase its incidence. Intestinal impaction with large amounts of sea urchins should be included in the differential diagnosis of gastrointestinal diseases in sea turtles, and the possible toxic effect of some sea urchin species on sea turtles should also be investigated.

## Introduction

Conservation medicine is a discipline that links animal health with ecosystem health and global environmental change [1]. Clinical and pathological studies on stranded sea turtles are essential activities for sea turtle conservation around the world. The loggerhead sea turtle (*Caretta caretta*) is the most common turtle species in the Canary Islands, and the North East Atlantic loggerhead subpopulation is included on the IUCN Red List of Threatened Species as ‘endangered’ [2].

Loggerhead diets are typically carnivorous, mainly comprised of mollusks and crustaceans that occur in neritic habitats, and to a lesser extent, fishes and cephalopods as discarded by-catch; sea urchins do not comprise an important part of their diet [3,4]. A variety of digestive diseases have been reported in loggerheads [5,6]. Some of them occur naturally, but other conditions, such as the digestive lesions associated with the ingestion of fishing hooks, fishing lines and crude oil, have an anthropogenic origin [5,7].

Mass extraction of fish for human use is a major stressor on marine ecosystems [8]. In the last decades, some coasts around the world have suffered an increase of the sea urchin populations, possibly due to the decrease of natural predators, mainly due to overfishing [9,10].

It has been recently mentioned the possibility of intestinal obstruction and perforation in loggerheads that fed extensively on pen shells (*Atrina* spp.) and sea urchins, but no additional data were provided by the authors [11]. The aim of this study was to describe the pathological and laboratory findings in a case series of stranding and mortality associated with ingestion of large amounts of sea urchins in loggerhead turtles.

## Materials and methods

### Ethics statement

Sea turtle rehabilitation program at the Tafira Wildlife Rehabilitation Center (TWRC) was conducted with authorization of the Wildlife Department of the Canary Islands Government (Ms. Guacimara Medina, Vice-chairman of Environmental Issues of the Canary Islands Government), and the Environment Department of the Cabildo de Gran Canaria (Ms. María del Mar Arévalo, Chairman of Environmental Issues of the Cabildo de Gran Canaria). Animal work and all sampling procedures were specifically approved as part of obtaining the authorization by the TWRC Animal Care Committee and the insular government Cabildo de Gran Canaria, and were consistent with standard vertebrate protocols and veterinary practices. No method of euthanasia was applied because the animals died naturally as a result of their lesions.

### Animals and study area

The six loggerheads studied were stranded on the coasts of Gran Canaria (27º 44’-28º 11’North, 15º 21’-15º 50’West), Canary Islands, Spain, between 2008 and 2015. Of these, three turtles had been submitted for health evaluation and possible rehabilitation, but died within the first hours. The mean ± standard deviation of the straight carapace length (SCL) and weight of the turtles were 66.5 ± 8.2 cm and 39.8 ± 3.2 kg, respectively. Based on SCL, all specimens were identified as sub-adult. The turtles stranded alive were lethargic and non-responsive to external stimuli.

### Methodology

Necropsies were performed at the Veterinary Faculty (ULPGC) using the procedures previously described [12,13]. Macroscopic lesions were recorded and tissue samples from all major organs were fixed in 10% neutral buffered formalin, embedded in paraffin, sectioned at 5 μm for light microscopy and stained with hematoxylin and eosin; special stains performed on selected samples also included Gram-stain for bacteria.

Samples from gross lesions and from spleen were cultured on a variety of selective and non-selective media (Oxoid Ltd, Basingstoke, UK), including blood agar, Mac-Conkey agar, Baird Parker agar for staphylococci, and Sabouraud Dextrose agar for fungi and yeasts. Bacteria were identified based on the biochemical profile (API 20 E, API 20 NE, and API 20 Staph, BioMérieux, Marcy-l'Étoile, France).

Sea urchin remains, including the pyramids that make-up part of Aristotle’s lantern, were cleaned, dried, measured and later photographed, in order to proceed with their correct identification using the procedures previously described [14].

## Results

**Fig 1:**
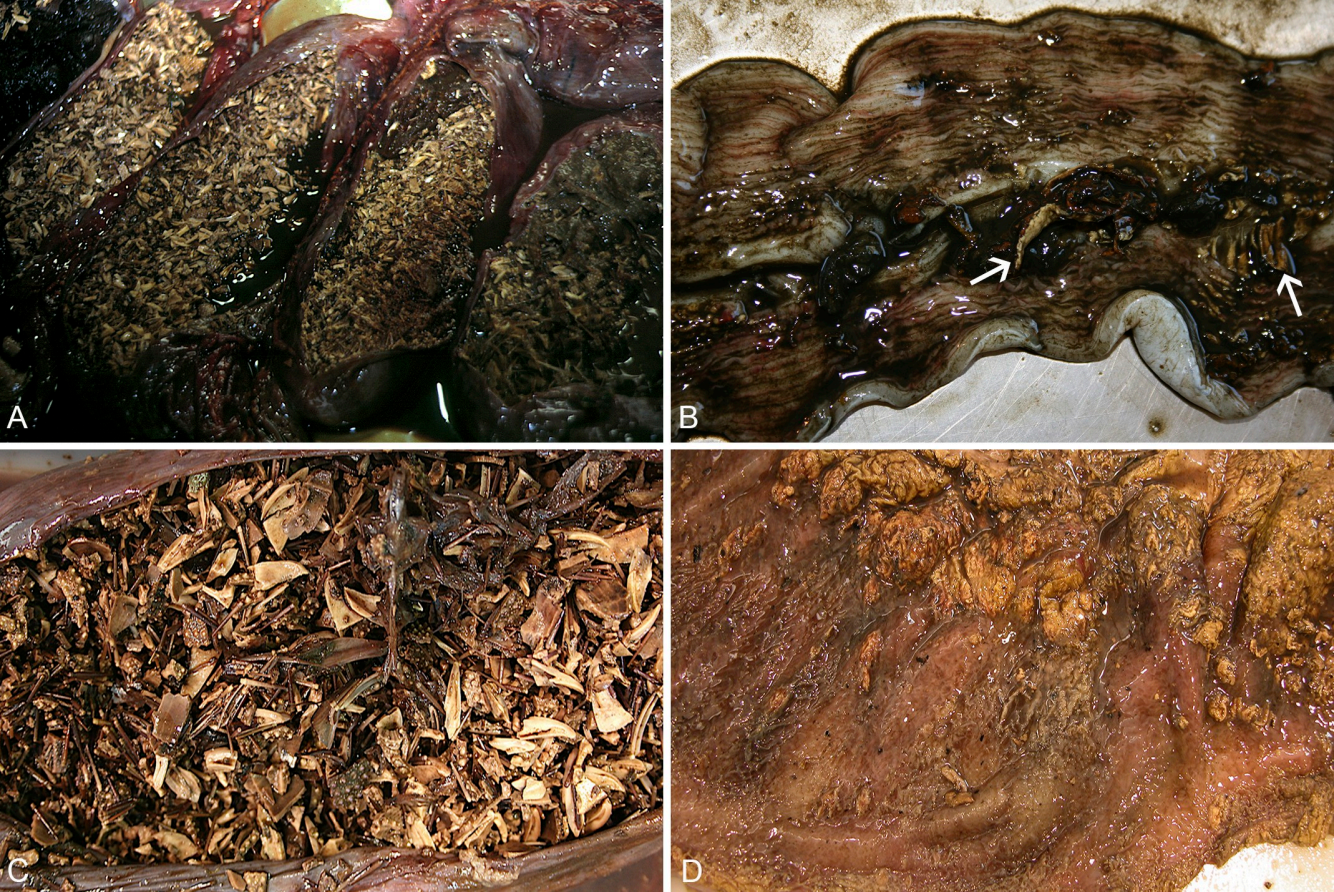
Gross findings in stranded loggerhead sea turtles caused by ingestion of large amounts of sea urchins. A) Severe colonic impaction, and B) deposits of fibrin (arrows) in the mucosal surface of the ileum of the same turtle; note also the edema of the intestinal wall. C) Severe intestinal (jejunum) impaction, and D) mucosa of the jejunum of the same turtle focally covered by deposits of fibrin.

At necropsy, all turtles showed good body condition with abundant amount of adipose tissue in the celomic cavity. No turtle showed lesions compatible with entanglement or other fishing gear interaction. All turtles showed severe intestinal impaction caused by large amounts of test fragments, broken spines, and pieces of feeding apparatus of sea urchins (2.1 ± 0.3 kg), mainly affecting the colon and the caudal half of the small intestine, whose mucosal surfaces were hyperemic. Two turtles showed deposits of fibrin, 2–5 cm in diameter, in areas of the small intestinal mucosa (Fig 1). The stomach and the complete small intestine of a turtle were also affected by the impaction. No perforations of the intestinal wall were observed. No gross lesions were visible in other organs. No parasites were detected in any of the turtles.

**Fig 2:**
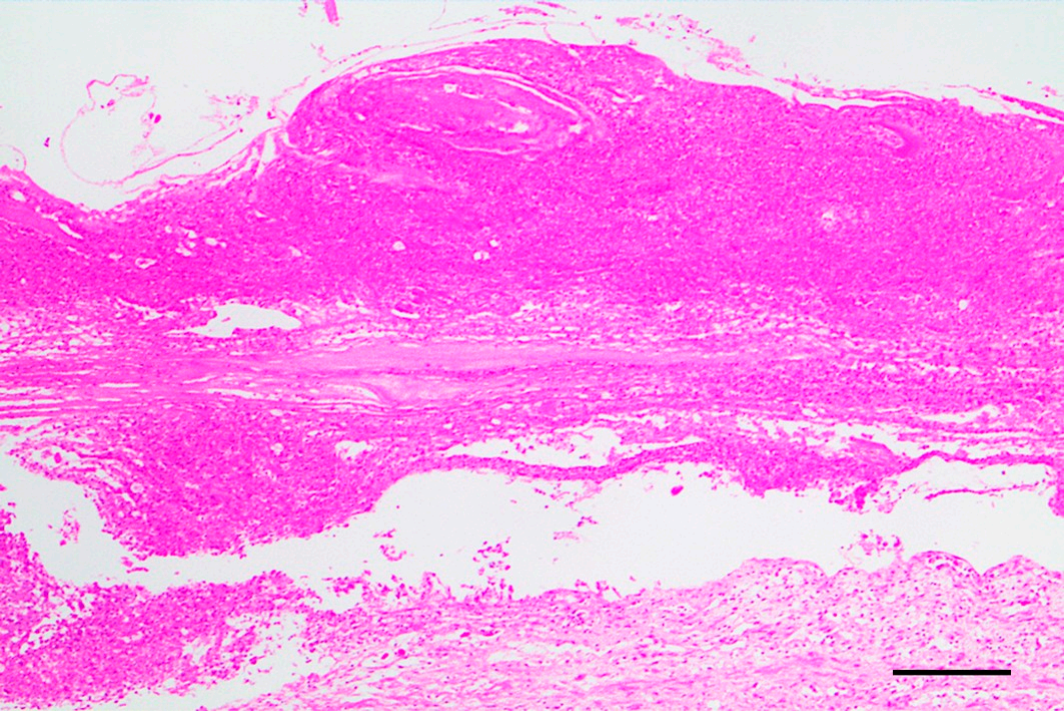
Severe focal fibrinonecrotic enteritis in the jejunum of a loggerhead sea turtle stranded due to ingestion of large amounts of sea urchins. HE. Bar = 500 μm.

Histologically, severe focal fibrinonecrotic enteritis was diagnosed in two turtles (Fig 2). In the remaining turtles, lesions ranged from mild desquamation of the intestinal epithelium to severe congestion of the blood vessels of lamina propria, submucosa, muscular and serosa, and edema. No histological lesions were detected in other major organs.

*Vibrio* sp. was isolated from the spleen and intestinal mucosa of a loggerhead in which focal fibrinonecrotic enteritis had been diagnosed.

**Fig 3:**
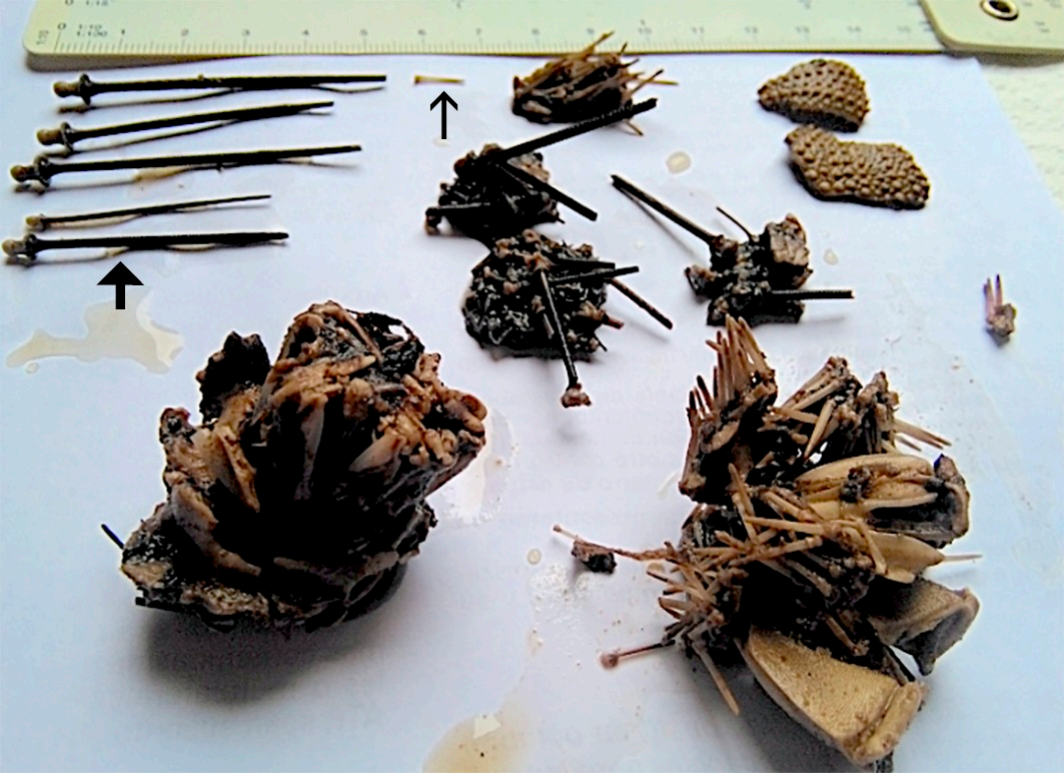
Sea urchin remains obtained from the intestine of a stranded loggerhead sea turtle. Note the fragments of tests with pieces of feeding apparatus (Aristotle’s lantern) and spines. Long black spines (thick arrow) belong to *Diadema africanum* whereas small white spines (thin arrow) are from *Sphaerechinus granularis*.

In five turtles, all the remains were fragments of tests and black spines, 4–5.5 cm long, from long-spined sea urchins (*Diadema africanum*). The last turtle contained a mixture of long-spined sea urchin (90%) and purple sea urchin (*Sphaerechinus granularis*) (10%) remains (Fig 3).

## Discussion

Although digestive diseases are frequently diagnosed in stranded sea turtles, there are no detailed reports of mortality associated with ingestion of sea urchins in sea turtles. Since anthropogenic causes are frequently reported, gastrointestinal obstruction is well documented for cases of ingestion of plastic bags or fishing nets [15]. Other causes, dietary in origin, are less frequently observed, and often are associated with diminished nutritional condition and underlying motility or ileus [11].

Gastrointestinal foreign bodies are commonly diagnosed in wild and captive marine turtles [16]. Wild turtles often swallow rocks, stones, coral and shells while foraging, and the presence of these objects alone, commonly seen upon radiographic evaluation, is not necessarily associated with disease [17]. It has been suggested that under certain poorly defined conditions, such as immunosuppression or altered motility of the gastrointestinal tract, these objects may contribute to complete or partial obstruction of the intestine [16]. It can also be difficult to determine if the presence of foreign material in sick turtles is a primary cause of illness or if other factors such as infection-related gastrointestinal stasis are present [16].

Health status of free-living sea turtle species are usually evaluated using the epidemiological data of turtles admitted to wildlife rehabilitation hospitals [7,18]. In a recent study on causes of stranding and mortality of loggerheads in Gran Canaria (1998–2014), the most frequent causes of admission for 392 sub-adult specimens were ingestion of hooks and monofilament lines (36%), entanglement in fishing gear and/or plastics (33.4%), and unknown/undetermined (14.8%). The other primary causes (trauma, infectious disease, crude oil, other causes) had frequencies below 9% [7]. Our results indicate that the incidence of mortality of sub-adult loggerheads due to ingestion of sea urchins is less than 1.6%. In addition, we detected 7 more cases of stranding possibly attributed to the ingestion of smaller amounts of sea urchins, although after the elimination of these remains of sea urchin tests and spines in the feces during the hospitalization period, the turtles were released.

Several authors suggested sea urchins as an increasing component in the loggerhead turtles diet, due to the lack of usual preys, which is a direct cause of overfishing [3,19]. Since loggerheads are not high-speed predators, they usually have an opportunistic behavior [3]. The increase of sea urchins in some areas can also be explained by the reduction of their natural predators due to the overfishing and the rise of the average temperature of the coastal waters [20]. Therefore, overfishing can have a double consequence: it can cause an increase in the populations of sea urchins due to the reduction of their natural predators, and, on the other hand, it reduces the availability of usual sea turtle preys, inducing them to consumption of unusual preys such as sea urchins.

Regarding the pathogenesis, in cases where the large intestine is affected by impaction, death is primarily associated with absorption of bacterial toxins through the intestinal wall and subsequent toxemia, and water and electrolyte balance are not severely affected [16]. In our study, fibrinonecrotic enteritis was only diagnosed in two turtles, and bacteremia caused by *Vibrio* sp. was detected only in one of them; however, no histological lesions were detected in the spleen from which *Vibrio* sp. was isolated. The latter together with the mild lesions observed in the remaining turtles and the general good body condition of all the turtles suggest that the disorder was acute.

Unfortunately, early diagnosis was not possible in the turtles stranded alive due to the death of the animals within the first hours. Diagnostic procedures, including contrast radiography, ultrasound, and celioscopy, can provide early diagnosis for intestinal impactions [11,16]. Computed tomography and magnetic resonance imaging are less frequently used because are less available and more expensive techniques [11]. Medical therapy using white petrolatum and light mineral oil has been reported to manage an intestinal partial obstruction caused by rubber items in a loggerhead turtle [21].

Some sea urchin species are venomous, but no fatal interaction with sea turtles has been reported. The hollow primary spines of some diadematid sea urchins are suggested to contain bioactive substances, and the toxopneustid sea urchins have globiferous pedicellaria with pharmacologically-active substances [22,23]. No studies about possible bioactive substances from *Diadema africanum* have been reported. However, the venom apparatus of the globiferous pedicellaria of the toxopneustid *Sphaerechinus granularis* has been described [24]. In the case of the turtle with a mixture of sea urchin species, a possible toxic effect added to the effects of intestinal impaction can’t be ruled out.

*Vibrio alginolyticus* has been associated with mass mortality of *Diadema africanum* in the Canary Islands [25]. In our case, health status of the ingested sea urchins was not investigated. Bacteremia caused by *Vibrio* sp. was detected only in a turtle, in which focal fibrinonecrotic enteritis had been diagnosed. Although we can’t rule out that the origin of the infection were the ingested sea urchins, *Vibrio* sp. is very frequent in the marine water and cloaca of apparently healthy sea turtles, being also able to enter to the blood vessels from the intestinal lesions [26].

In conclusion, although the prevalence of this cause of stranding was low compared to other mortality causes, continued overfishing and anthropogenic climate change could increase its incidence. In addition, intestinal impaction with large amounts of sea urchins should be included in the differential diagnosis of gastrointestinal diseases in sea turtles, particularly in sub-adult specimens. Finally, the possible toxic effect of some species of sea urchins on sea turtles should be investigated.

## Supporting information

S1 File Data of loggerhead sea turtles included in the survey. (XLSX)
